# Increased Corneal Epithelial Turnover Contributes to Abnormal Homeostasis in the *Pax6^+/−^* Mouse Model of Aniridia

**DOI:** 10.1371/journal.pone.0071117

**Published:** 2013-08-13

**Authors:** Panagiotis Douvaras, Richard L. Mort, Dominic Edwards, Kanna Ramaesh, Baljean Dhillon, Steven D. Morley, Robert E. Hill, John D. West

**Affiliations:** 1 Centre for Integrative Physiology, University of Edinburgh, Edinburgh, United Kingdom; 2 Tennent Institute of Ophthalmology, Gartnaval General Hospital, Glasgow, United Kingdom; 3 School of Clinical Sciences, University of Edinburgh, Edinburgh, United Kingdom; 4 Division of Health Sciences, University of Edinburgh, Edinburgh, United Kingdom; 5 Medical Research Council Human Genetics Unit, Medical Research Council Institute of Genetics and Molecular Medicine, University of Edinburgh, Edinburgh, United Kingdom; University of Reading, United Kingdom

## Abstract

We aimed to test previous predictions that limbal epithelial stem cells (LESCs) are quantitatively deficient or qualitatively defective in *Pax6^+/−^* mice and decline with age in wild-type (WT) mice. Consistent with previous studies, corneal epithelial stripe patterns coarsened with age in WT mosaics. Mosaic patterns were also coarser in *Pax6^+/−^* mosaics than WT at 15 weeks but not at 3 weeks, which excludes a developmental explanation and strengthens the prediction that *Pax6^+/−^* mice have a LESC-deficiency. To investigate how Pax6 genotype and age affected corneal homeostasis, we compared corneal epithelial cell turnover and label-retaining cells (LRCs; putative LESCs) in *Pax6^+/−^* and WT mice at 15 and 30 weeks. Limbal BrdU-LRC numbers were not reduced in the older WT mice, so this analysis failed to support the predicted age-related decline in slow-cycling LESC numbers in WT corneas. Similarly, limbal BrdU-LRC numbers were not reduced in *Pax6^+/−^* heterozygotes but BrdU-LRCs were also present in *Pax6^+/−^* corneas. It seems likely that *Pax6^+/−^* LRCs are not exclusively stem cells and some may be terminally differentiated CD31-positive blood vessel cells, which invade the *Pax6^+/−^* cornea. It was not, therefore, possible to use this approach to test the prediction that *Pax6^+/−^* corneas had fewer LESCs than WT. However, short-term BrdU labelling showed that basal to suprabasal movement (leading to cell loss) occurred more rapidly in *Pax6^+/−^* than WT mice. This implies that epithelial cell loss is higher in *Pax6^+/−^* mice. If increased corneal epithelial cell loss exceeds the cell production capacity it could cause corneal homeostasis to become unstable, resulting in progressive corneal deterioration. Although it remains unclear whether *Pax6^+/−^* mice have LESC-deficiency, we suggest that features of corneal deterioration, that are often taken as evidence of LESC-deficiency, might occur in the absence of stem cell deficiency if corneal homeostasis is destabilised by excessive cell loss.

## Introduction

The adult corneal epithelium is a constantly renewing tissue and it is widely accepted that, during normal homeostasis, it is maintained by a stem cell population in the basal limbal region that proliferates slowly unless stimulated by injury [1], [2]. These limbal epithelial stem cells (LESCs) give rise to fast-dividing transient (or transit) amplifying cells (TACs), which migrate centripetally in the basal layer of the corneal epithelium [3], [4], [5]. Here they proliferate for a limited time before undergoing a final division, whereupon both daughter cells usually detach from the basement membrane, move vertically (apically) through the suprabasal layers, becoming terminally differentiated and are eventually shed from the most superficial layer [6], [7].

The absence of reliable markers, able to distinguish adult stem cell populations from early TACs in the corneal epithelium, means that various indirect methods have been used to deduce that the basal limbal epithelium is the niche for corneal epithelial stem cells. Two threads of information from mouse studies have been important: the demonstration of centripetal migration of corneal keratinocytes from the limbus towards the central cornea [4], [5] and the identification of putative stem cells as slow cycling ‘label-retaining cells’ (LRCs).

Early studies revealed that a characteristic feature of epithelial stem cells is that they divide relatively infrequently [8] and a widely held hypothesis is that stem cells are generally slow cycling during normal homeostasis but they can be induced to proliferate faster after injury. Dividing cells can be labelled by incorporating a label into the DNA (e.g. bromodeoxyuridine, BrdU) and to ensure slow cycling cells are labelled, the animals can be exposed to the label for a prolonged period. This is followed by an extended chase period, which dilutes the label more quickly in more rapidly dividing cells so revealing slow-cycling putative stem cells by their ability to retain the label. In the wild-type (WT) ocular surface, LRCs are found in the basal layer of the conjunctival and limbal epithelia, whereas the corneal epithelium is usually devoid of such slow-cycling cells [2], [6], [9], [10], [11], [12], [13].

Human aniridia is an inherited eye disease caused by heterozygosity for a defective *PAX6* gene. The phenotype involves developmental eye abnormalities, including a reduced or absent iris, [14], [15], [16], [17], and postnatal corneal deterioration known as aniridic keratopathy or aniridia-related keratopathy (ARK) [18], [19], [20]. The mouse *Pax6^Sey-Neu^* mutant allele is considered to be a *Pax6^−^* null allele and heterozygous *Pax6^+/Sey-Neu^* (here abbreviated to *Pax6^+/−^*) mice have small eyes, hypoplastic irides and a range of other ocular abnormalities, including corneal deterioration, so provide a good model for human *PAX6^+/−^* aniridia and ARK [21]. Some mouse *Pax6^+/−^* corneal abnormalities arise during development (e.g. the corneal epithelium is already thinner than normal by embryonic day 18.5 (E18.5) [21]) whereas other abnormalities arise during adulthood (e.g. blood vessels invade the corneal stroma, goblet cells accumulate in the corneal epithelium and centripetal epithelial cell movement is disrupted) [21], [22].

The corneal epithelial deterioration seen in *PAX6^+/−^* humans and *Pax6^+/−^* mice can be considered to represent abnormal tissue homeostasis. From a purely quantitative perspective, tissue homeostasis can be defined as the maintenance of an approximately constant cell number in a renewing tissue and so involves a balance of cell production and cell loss. However, to conserve full tissue functionality it is critical that lost cells are replaced by cells of the appropriate phenotype. Thus, tissue homeostasis can be considered to have both quantitative and qualitative aspects. Normal corneal epithelial homeostasis requires maintenance of an adequate number of cells of the phenotype required to ensure the cornea is able to maintain full transparency and an adequate barrier function. The accumulation of goblet cells that occurs during the deterioration of the corneal epithelia in *PAX6^+/−^* humans and *Pax6^+/−^* mice implies that homeostasis is qualitatively abnormal. This could arise either because corneal epithelial cell differentiation is abnormal or because conjunctival or other cells encroach onto the corneal surface to compensate for a numerical deficiency in corneal epithelial cells caused by reduced cell production and/or excessive cell loss.

**Figure 1:**
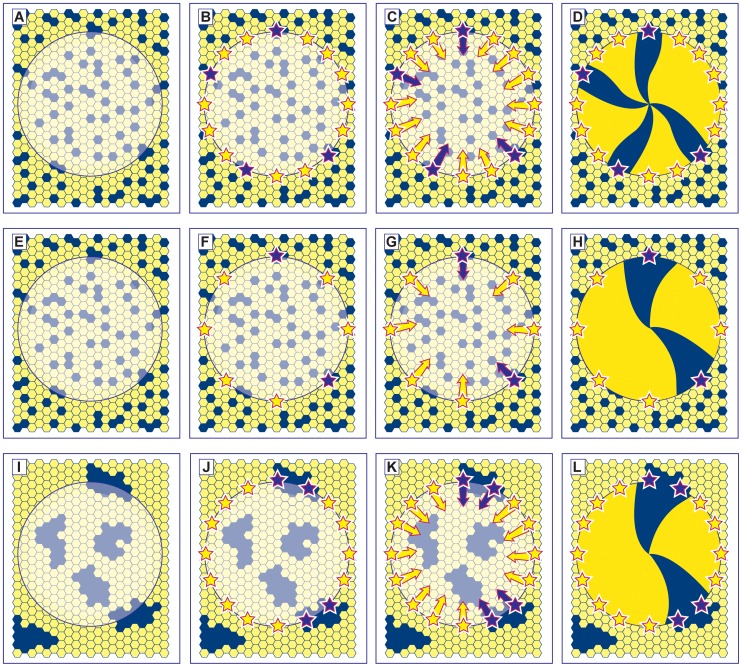
Alternative hypotheses to explain the reduction in corrected stripe numbers in *Pax6^+/−^* mosaic corneas. **(A–D) Normal development:** The developing mosaic surface ectoderm (A) comprises two genetically marked cell populations (shown as blue and yellow hexagons) and the future corneal epithelium is shown as a disk with the limbus at its periphery. LESCs are specified (shown as stars in B) from a pool of cells in the surface ectoderm and become active postnatally (C). Centripetal movement of daughter TACs forms radial stripe patterns in the adult corneal epithelium (D). **(E–H) Hypothesis 1– reduced** ***Pax6^+/−^*** **LESC numbers:** If fewer LESCs are specified in the *Pax6^+/−^* ocular surface (F) then individual clones of corneal epithelial cells produced by each LESC will colonise a larger sector of the cornea, forming fewer, wider stripes (H). A similar result is expected if normal numbers of LESCs are specified but some fail to survive. **(I–L) Hypothesis 2– reduced cell mixing during** ***Pax6^+/−^*** **development:** If there is less mixing of the two genetically marked cell populations in the *Pax6^+/−^* mosaic surface ectoderm during development, cells will be grouped into larger coherent clones to form a coarse-grained mosaic pattern (I). There is a higher probability that two adjacent stem cells belong to the same population (e.g. adjacent blue stem cells in J) so wider stripes are produced. Although, in this case, the distribution of LESCs around the circumference will be non-random, the distribution of LESC clones should still be random. The corrected stripe number described in the text is expected to be proportional to the number of LESC clones but the number of LESCs per clone may vary as shown (e.g. compare H and L).

It has been proposed that LESC deficiency is the principal cause of deterioration of the ocular surface in aniridia patients [20], [23]. Indirect evidence from analysis of coherent clonal lineages in adult mouse X-inactivation mosaics, transgenic mosaics and chimeras predicts that LESCs are also either numerically deficient and/or functionally defective in heterozygous *Pax6^+/−^* mice [22] and older WT mice [24], [25], [26]. These mice exhibit radial stripes, which are thought to represent clonally related populations of corneal keratinocytes migrating from a LESC population at the periphery. *Pax6^+/−^* X-inactivation mosaic corneas had fewer radial stripes than WT mosaic corneas at 15 weeks [22]. This was assumed to imply either that LESCs were reduced in number or qualitatively defective but this could also be explained by an alternative hypothesis. The diagrams in Fig. 1 represent production of radial corneal stripes after LESC activation in the WT ocular surface (Fig. 1A–D) and illustrate two hypotheses, which could account for why *Pax6^+/−^* mosaic corneas have fewer, wider stripes. Hypothesis 1 (Fig. 1E–H) proposes that fewer *Pax6^+/−^* LESCs are initially specified during embryonic development (or some LESCs fail to survive), so there is a stem cell deficiency. Hypothesis 2 (Fig. 1I–L) proposes that the extent of cell mixing during development is less in the *Pax6^+/−^* ocular surface than in WT, so the mosaicism is more coarse-grained (with larger coherent clones) and clonally related LESCs are more likely to be adjacent to each other. It is important to evaluate whether cell mixing is reduced during development (hypothesis 2) before drawing the conclusion that the mosaic analysis predicts that LESC function is impaired in *Pax6^+/−^* mice (hypothesis 1).

Other factors also probably contribute to abnormal corneal homeostasis in *Pax6^+/−^* heterozygotes. Two observations suggest that cell turnover is more rapid in the corneal epithelium. First, the frequency of BrdU labelled cells has been reported to be higher in *Pax6^+/−^* mice than WT mice, either for the whole corneal epithelium [27] or specifically the basal layer [28]. Second, *ex vivo* evidence suggests that the *Pax6^+/−^* corneal epithelium is more fragile than normal [27]. This is consistent with observed abnormalities in cell adhesion molecules, junctional complex proteins and actin-based cytoskeletal structures plus the reduced expression of keratin 12 (K12) [27], [28], [29]. K12 is a specific marker of corneal epithelial differentiation that is regulated by Pax6 [30], [31], [32], so its reduced expression in *Pax6^+/−^* corneal epithelia suggests that differentiation of the *Pax6^+/−^* corneal epithelium is abnormal [27], [28]. Down-regulation of K12 also probably contributes to *Pax6^+/−^* epithelial fragility, as reported for K12 knockout mice [33]. This increased fragility prompted the proposal that the *Pax6^+/−^* cornea is in a chronic wound-healing state [29], [34]. These observations all predict that *Pax6^+/−^* corneal epithelial cell loss would be greater than normal but this has not been investigated directly.

The aims of the present study were: (1) to compare the rate of movement of cells from the basal corneal epithelium to the suprabasal layers in *Pax6^+/−^* and WT mice, as this is the irreversible first step of cell loss; (2) to re-evaluate the prediction from studies of mosaic mice that *Pax6^+/−^* mice have fewer LESCs by testing whether the coarser mosaic patterns could be accounted for by reduced cell mixing during development of the *Pax6^+/−^* ocular surface, so producing larger coherent clones (hypothesis 2 shown in Fig. 1I–L); (3) to compare the number of label-retaining cells (LRCs) in the limbus of *Pax6^+/−^* and WT mice at two ages (15 and 30 weeks), as a means of comparing relative LESC numbers, to test whether *Pax6^+/−^* mice have fewer LESCs than WT (hypothesis 1 shown in Fig. 1E–H) and whether LESC numbers decline with age in WT mice.

## Materials and Methods

### Ethics Statement

All the animal work in this study was approved by the University of Edinburgh Ethical Review Committee (application PL21-06) and performed in accordance with UK Home Office regulations under project license number PPL 60/3635. All surgery was performed under general anaesthesia and all efforts were made to minimise suffering.

### Mice

Heterozygous *Pax6^+/Sey-Neu^* (*Pax6^+/−^*) and *Pax6^+/+^*, wild-type (WT) littermates were maintained on a congenic CBA/Ca genetic background. They were distinguished by eye size and their genotypes confirmed by PCR [35]. Transgenic H253 mice [36], which ubiquitously express the X-linked *Tg(Hmgcr-LacZ)H253Sest, nLacZ* transgene (abbreviated to *XLacZ*), were maintained on a mixed genetic background (predominantly C57BL/6 and CBA/Ca). Hemizygous males and females are designated respectively *XLacZ^Tg/Y^* and *XLacZ^Tg/−^*. Both *Pax6^+/−^, XLacZ^Tg/−^* and WT, *XLacZ^Tg/−^* X-inactivation mosaics were produced from *Pax6^+/−^* female × *XLacZ^Tg/Y^* male crosses. Groups of mice were compared at 15 and 30 weeks because previous comparisons of WT X-inactivation mosaics showed that an age-related decline in corrected corneal epithelial stripe numbers could be detected between these ages [22], [24], [25], [37]. We, therefore, compared both corrected corneal epithelial stripe numbers and the numbers of limbal label-retaining cells in *Pax6^+/−^* and WT X-inactivation mosaics at 15 and 30 weeks, as described later. Differences between these ages are considered to be age-related differences but not effects of “old age” as laboratory mice can live to about 2 years [38]. Mice were bred and maintained in animal facilities at the University of Edinburgh and killed by cervical dislocation following inhalation of gaseous anaesthetic.

### BrdU Treatment

For acute BrdU labelling of the adult cornea, 12-week old mice were given single intraperitoneal (i.p.) injections of BrdU (10 mg BrdU/ml saline; 0.2 ml/mouse) at 10.00 am. Mice were killed after 4 hours or 1, 3, 7 or 14 days and samples collected. For BrdU pulse-chase identification of label-retaining cells (LRCs), 0.1 ml Alzet mini-osmotic pumps (model 1007D, Charles River, UK Ltd.), containing BrdU solution (0.1 ml; 50 mg BrdU/ml in saline) were surgically implanted, sub-cutaneously, under Isoflurane anaesthesia, into 15- or 30-week old mice to provide 0.5 µl BrdU solution/h. The pumps were removed after 7 days and mice were killed 10 weeks later and samples collected.

### BrdU Immunohistochemistry

Eyes were fixed in 4% paraformaldehyde for 24 h. and embedded in paraffin wax after dehydration in a graded ethanol series. Longitudinal sections were cut at 7 µm thickness, mounted on Polysine slides (VWR laboratory supplies), deparaffinised in Histoclear and processed for BrdU immunoshistochemistry with a 3-3′diaminobenzidine (DAB) endpoint and lightly counterstained with haematoxylin, as described previously [28]. However, for antigen retrieval, sections were immersed in citrate buffer (10 mM sodium citrate pH 6.0) in a covered Coplin jar in a 90°C water bath. Control slides were incubated with normal serum without the primary antibody but otherwise treated in the same way.

### Analysis of Cell Proliferation and Loss

After BrdU immunohistochemistry, calibrated digital images of mid-sections of the corneal epithelium were captured with a Nikon Coolpix 995 digital camera on a Zeiss Axioplan 2 compound microscope. Every basal corneal epithelial cell was scored as positive or negative, across the corneal diameter in a section from the middle of the eye. These data were divided into six equal sized groups according to corneal region, so the % BrdU-labelled nuclei in the basal layer (basal layer labelling index, LI) could be determined separately for two peripheral (P), intermediate (I) and central (C) regions which were then combined to provide one mean value per eye for the P, I and C regions. The % BrdU-positive cells in all the suprabasal layers (suprabasal LI) were scored in an equivalent way. The *Pax6^+/−^* corneal epithelium has fewer cell layers than WT and this could exaggerate or mask differences in suprabasal LI. To take account of this, an adjusted % BrdU-positive cells in the suprabasal layers (adjusted suprabasal LI) was calculated by expressing the number of BrdU-positive cells in the suprabasal layers as a percentage of the total cell number in the underlying basal layer rather than the total cell number in the suprabasal layers.

### Whole-mount Cornea Immunofluorescence

Whole-mount corneal immunofluorescence was adapted from a published method [39]. Eyes were dissected, washed with PBS, fixed in 4 methanol : 1 DMSO and rehydrated through a graded methanol series to PBS. Corneal buttons were dissected, incubated for 2 h. in blocking serum, comprising PBS-T (PBS containing 0.02% Tween20), 1% BSA and 1% goat serum. They were then incubated with primary antibody overnight at 4°C, washed (PBS-T; 5 h.), treated with blocking serum and incubated with secondary antibodies overnight at 4°C. Finally, corneas were washed (PBS-T; 4 h.), counterstained with TO-PRO3 iodide (Invitrogen) containing RNaseA, flattened with radial cuts and mounted on slides with MoWiol 4–88 (Calbiochem) containing 2.5% DABCO (1,4-diazobicyclooctane). For LRC analysis, each cornea was flat-mounted with 8 radial cuts, creating 8 corneal sectors.

The primary antibodies used were rat anti-BrdU (Abcam), rabbit anti-K19 (LifeSpan Biosciences) and rat anti-CD31 (BD Pharmingen). The secondary antibodies were goat Alexa 488 Anti-Rat IgG (Invitrogen), goat Alexa 488 Anti-Rabbit IgG (Invitrogen) and goat Alexa 568 Anti-Rat IgG (Invitrogen). For double immunofluorescent staining, antibodies were applied sequentially and incubated overnight at 4°C. To image K19 and CD31 immunofluorescence, overlapping Z-stack images were acquired with a Leica TCS NT confocal microscope and exported to ImageJ (http://rsb.info.nih.gov/ij/). Composite images were constructed using the MosaicJ plug-in (http://bigwww.epfl.ch/thevenaz/mosaicj). BrdU immunofluorescence analysis is described below.

### Analysis of BrdU Label-retaining Cells in Whole Mount Corneas

Eight Z-stack images, comprising 5–6 optical sections at 2–3 µm intervals that included the limbus, were acquired per cornea (1 for each of 8 sectors), in a clockwise direction with a Zeiss LSM510 CLSM confocal microscope using a ×20 lens. Images (with the limbus in the middle) were acquired at 1024×1024 pixel resolution. Individual photographs were compiled using Zeiss LSM Image Browser software (version 4.2.0.121) and projected to a single image based on maximum intensities. ImageJ was used for thresholding, to include particle sizes of 150–5000 voxels (3–15 µm diameter nuclei). A calibrated 340×200 µm sampling box was superimposed on the image across the width of the limbal region in each sector and BrdU-positive nuclei were counted. Fluorescent spots outside the size range for BrdU-positive nuclei were not included in the analysis.

Counts of BrdU-positive nuclei were expressed as the mean number of LRCs per sampling box (one sampling box per sector). To correct for differences in corneal diameters, the mean number of LRCs per sampling box was multiplied by the corneal circumference (estimated from 3 diameter measurements of the flattened cornea) and divided by the length of the sampling box (340 µm) to provide an estimate of the number of LRCs in a 200 µm wide ring around the circumference.

To reduce the effect of variability among individual immunofluorescence experiments, the mean number of LRCs per sampling box was also expressed as a normalised ‘LRC index per sampling box’. This was calculated separately for each immunofluorescence experiment that yielded results for at least one eye from each of the four groups compared. First, to accommodate experiments with unequal number of eyes in each group, a normalised total number of LRCs was calculated for each experiment as the sum of the mean number of LRCs per sampling box for each of the four groups. The ‘LRC index per sampling box’ was then calculated separately for each of the four groups as the number of LRCs per sampling box, expressed as the percentage of the normalised total number of LRCs per sampling box (for all four groups) for each experiment. The sum of the LRC indices for the four groups is, therefore, equal to 100% in each experiment. The estimated number of LRCs per circumference was similarly expressed as the ‘LRC index per circumference’.

The confocal corneal images were also used to compare cell-packing densities in the four groups, using 3 regions from each of 3 corneas per group. Nuclei were counted manually in a 150×150 µm sampling box that was superimposed on the image by an independent person using Adobe Photoshop 7.0.

### β-galactosidase Histochemistry and Analysis of Mosaic Patterns in Histological Sections

Whole eyes from *XLacZ^Tg/−^*, X-inactivation mosaic mice were stained with X-gal as described elsewhere [24], [25]. For analysis of patch sizes at 3–10 weeks, the stained eyes were processed for routine paraffin wax histology and sections were counterstained in neutral red and eosin and mounted in DPX under coverslips. Cells in the basal layer of the mid-section of the corneal epithelium were counted and scored as β-gal-positive (stained blue) or β-gal-negative (red counterstain) across the diameter of the cornea by microscopy using a ×40 objective and the numbers of cells in alternate β-gal-positive and β-gal-negative patches were recorded. The number of patches, mean patch length and median patch length were recorded separately for the β-gal-positive and β-gal-negative cell populations and the % β-gal-positive cells was calculated. The total numbers of basal cells and patches across the corneal diameter were also recorded.

### Analysis of Stripe Numbers in the Corneal Epithelium of Adult X-inactivation Mosaics

Whole adult eyes were stained with X-gal and the radial stripe patterns in the corneal epithelia were analysed semi-automatically from digital photographs showing the entire cornea, using the ImageJ plugin ‘Clonal Tools’ in batch mode as described previously to provide a ‘corrected stripe number’ [37], [40]. This corrects for the probability that stripes would contain multiple adjacent β-gal-positive corneal epithelial clones as described previously [24], [25]. The corrected stripe number was also divided by the circumference of each cornea measured to allow for differences in eye sizes.

### Statistical Analysis

GraphPad Prism software was used for statistical tests as indicated in the text and figure legends. The choice of parametric or non-parametric tests was guided, in part, by normality tests. In some cases, data (or the data plus 1, to accommodate zero values) were log-transformed for analysis with parametric statistical tests. The error bars in the figures are 95% confidence intervals (CI).

## Results

### Cell Proliferation in the Basal Corneal Epithelium in WT and *Pax6^+/−^* Mice

**Figure 2:**
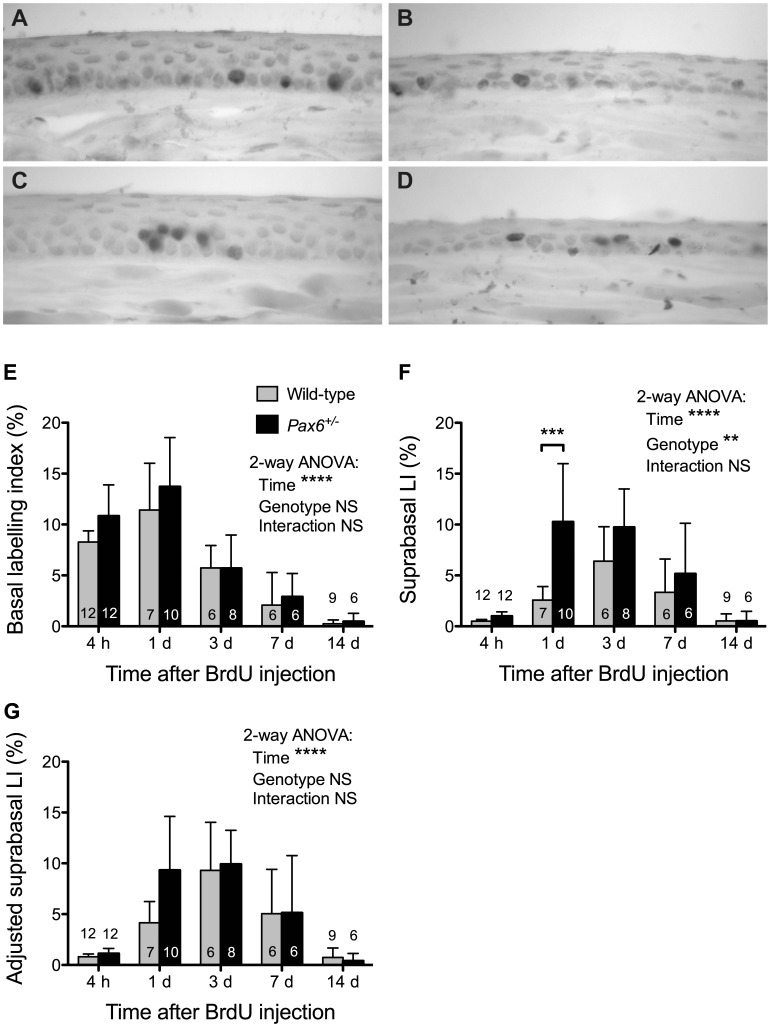
Acute BrdU labelling of WT and *Pax6^+/−^* corneal epithelia. (**A, B**) BrdU immunohistochemistry 4 hours after BrdU injection of (A) WT and (B) heterozygous *Pax6^+/−^* mice. (**C, D)** BrdU immunohistochemistry 24 hours after BrdU injection of (C) WT and (D) *Pax6^+/−^* mice. BrdU-positive nuclei in the corneal epithelium appear dark. (**E–G**) The mean (±95% CI) BrdU labelling indices for mid-sections are shown for chase periods of 4 hours (4 h) to 14 days (14 d). (**E**) BrdU basal labelling index (BrdU positive basal cells as a percentage of total basal cells). (**F**) BrdU suprabasal labelling index (BrdU positive suprabasal cells as a percentage of total suprabasal cells). (**G**) Adjusted suprabasal BrdU labelling index (BrdU positive suprabasal cells as a percentage of total basal cells). Results for 2-way analyses of variance (ANOVAs) for log transformed data are shown. Where genotype differences were significant overall, pairwise comparisons were made between genotypes for each time point using Bonferroni post-hoc tests (significant differences are shown by asterisks). Separate 1-way ANOVAs and Bonferroni post-hoc tests for each genotype showed that the frequencies of BrdU-positive cells increased in the suprabasal layers from 4 h to 3 days (*P*<0.001 for both WT and *Pax6^+/−^* in E & F) and then declined from 3 to 14 days (*P*<0.001 for both WT and *Pax6^+/−^* in E & F). Abbreviations: LI, labelling index; NS, not significant; **P<0.01; ***P<0.001; ****P<0.0001. 6–12 eyes per group as shown within or above the bars.

**Figure 3:**
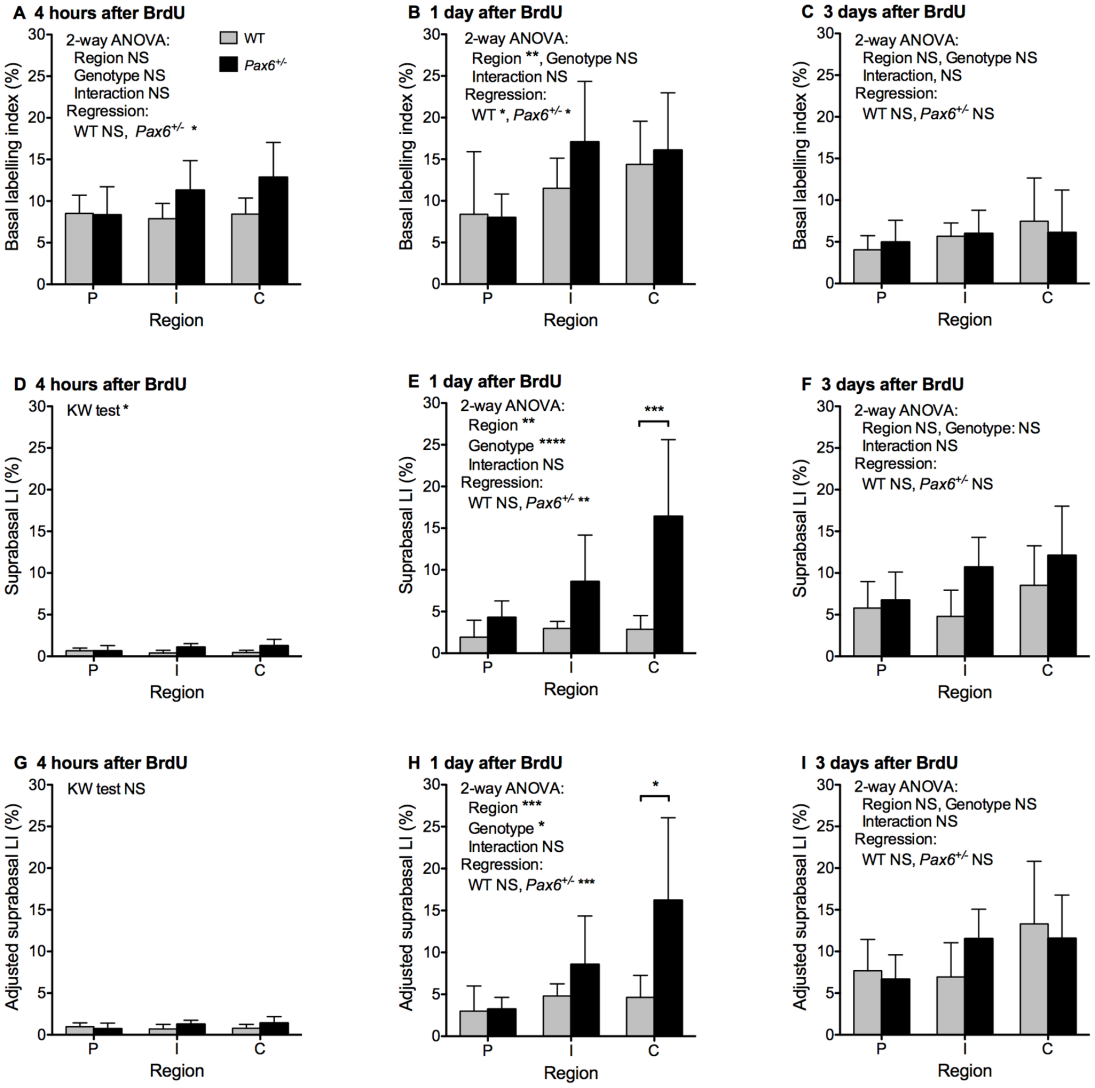
Distributions of BrdU-positive cells in different regions of WT and *Pax6^+/−^* corneal epithelia. The mean (±95% CI) BrdU labelling indices are shown separately for the peripheral (P), intermediate (I) and central (C) regions of the cornea for chase periods of 4 hours (A, D & G), 1 day (B, E & H) and 3 days (C, F & I). (**A–C**) BrdU basal labelling index (BrdU positive basal cells as a percentage of total basal cells). (**D–F**) BrdU suprabasal labelling index (BrdU positive suprabasal cells as a percentage of total suprabasal cells). (**G–I**) Adjusted suprabasal BrdU labelling index (BrdU positive suprabasal cells as a percentage of total basal cells). In most cases statistical comparisons were made by 2-way analyses of variance (ANOVAs) of log-transformed data followed by pairwise Bonferroni post-hoc tests and separate linear regression analyses for WT and *Pax6^+/−^* genotypes. Non-parametric Kruskal-Wallis (KW) tests followed by pairwise Dunn’s multiple comparison tests were used for D and G because there were many zero values and the log-transformed data were not normally distributed. Significant differences for the 2-way ANOVAs and linear regressions (or Kruskal-Wallis tests) are shown in each panel. The only two significant pairwise post-hoc tests between genotypes are shown by asterisks over the two bars compared (central region in E and H). The post-hoc tests between regions are not shown on the histograms. For WT corneas, only the post-hoc test between regions P vs. C in B was significant (*P*<0.05). For *Pax6^+/−^* corneas, post-hoc tests between pairs of regions were significant for P vs. C in A (*P*<0.05), B (*P*<0.05), E (*P*<0.001) and H (*P*<0.001), and for P vs. I in B (*P*<0.05) and H (*P*<0.05). Abbreviations: LI, labelling index; WT, wild-type; NS, not significant; *P<0.05; **P<0.01; ***P<0.001; ****P<0.0001. 6–12 eyes per group as shown in Fig. 2E–G.

The 12-week, WT corneal epithelium comprised 4–6 layers and the mid sections contained 424±10.1 basal cells (mean ±95% CI) and 669±21.9 broader and flatter suprabasal cells. *Pax6^+/−^* corneas were smaller in diameter (305±10.6 basal cells) and had fewer suprabasal layers (307±10.7 suprabasal cells across the diameter). As expected, BrdU-positive cells were almost entirely confined to the basal layer four hours after labelling (Fig. 2). The percentage of BrdU-positive cells in the basal corneal epithelial cell layer (basal labelling index, LI) increased slightly (but not significantly) between 4 and 24 hours in both *Pax6^+/−^* and WT corneas and then declined (Fig. 2E). There were also some significant differences in basal BrdU labelling among corneal epithelial regions, most notably at 1 day (Fig. 3A–C and legend). Although the basal LI was higher in *Pax6^+/−^* than WT eyes at both 4 and 24 hours after BrdU injection (Fig. 2E) as reported previously [28], the genotype differences did not reach statistical significance in the present experiment either when compared in whole corneal epithelium (Fig. 2E) or individual regions (Fig. 3A–C).

### Basal Corneal Epithelial Cells Move More Quickly to the Suprabasal Layers in *Pax6^+/−^* Mice

As *Pax6^+/−^* corneal epithelia had fewer suprabasal layers than WT, an ‘adjusted suprabasal LI’ was calculated as well as the suprabasal LI, to compare the percentage of BrdU-positive cells in the suprabasal layers (see Materials and Methods). The frequencies of BrdU-positive cells increased significantly in both WT and *Pax6^+/−^* suprabasal layers during the first three days and then declined (Fig. 2F,G). The initial increase in suprabasal BrdU labelling occurred more rapidly in *Pax6^+/−^* corneas and the suprabasal LI was significantly higher in *Pax6^+/−^* than WT eyes at 1 day (Fig. 2F), suggesting that more BrdU-labelled cells had moved from the basal layer to the suprabasal layers in *Pax6^+/−^* corneas. The adjusted suprabasal LI showed a similar trend at 1 day but the difference between genotypes was not significant (Fig. 2G). However, at this time the percentage of labelled cells in the suprabasal layers was significantly higher in *Pax6^+/−^* corneas, with or without adjustment for fewer suprabasal layers in *Pax6^+/−^* corneas (Table 1). Thus, the greater accumulation of labelled cells in *Pax6^+/−^* suprabasal layers is not simply a consequence of more labelled cells in the basal layer or fewer suprabasal layers and implies that cells move to the suprabasal layers more frequently in *Pax6^+/−^* corneas.

**Table 1:** Percentage of all the labelled cells that are in the suprabasal layers 24 hours after BrdU injection.

Calculation of % of labelled cellsthat are in the suprabasal layers	Mean Percentage ± SEM (N)		*P*-value
	Wild-type	*Pax6^+/−^*	
SLI ×100/(SLI+BLI)	18.23±1.64 (7)	40.03±3.93 (10)	*P* = 0.0003
ASLI ×100/(ASLI+BLI	26.20±1.93 (7)	38.01±3.72 (10)	*P* = 0.0144

*Abbreviations:* ASLI = adjusted suprabasal labelling index; BLI = basal labelling index; SLI = suprabasal labelling index. *P*-value is for *t*-test with Welch’s correction for unequal variances.

### *Pax6^+/−^* Basal Cells Move More Quickly to the Suprabasal Layers in the Central Corneal Epithelium

Differences in suprabasal BrdU LI between regions and genotypes were only significant at 1 day after BrdU injection (Fig. 3D–F and legend). Regional differences were confined to *Pax6^+/−^* corneas, which showed a much higher suprabasal LI in the central cornea than the periphery (*P*<0.001; Fig. 3E and legend). This was confirmed by linear regression analysis among regions, which was significant for *Pax6^+/−^* (*P*<0.01) but not WT corneas. At this time the suprabasal LI in the central cornea was significantly higher for *Pax6^+/−^* than WT (*P*<0.001; Fig. 3E). The results indicate that movement of basal cells to the suprabasal layers occurs more rapidly in *Pax6^+/−^* than WT corneas and in *Pax6^+/−^* corneas it occurs most rapidly in the central region. The adjusted suprabasal LIs followed a similar trend to the unadjusted suprabasal LIs (Fig. 3G–I). It varied significantly with region and *Pax6* genotype at 1 day (Fig. 3H and legend) and again genotype differences were significant only for the central region at 1 day (*P*<0.05; Fig. 3H).

As the basal to suprabasal cell movement is irreversible and is the first step in a sequence of events that culminates in cell loss from the corneal epithelial surface, the results shown in Fig. 3E,H imply that cell loss is greater from corneas of *Pax6^+/−^* than WT mice and that this difference is most pronounced in the central region of the cornea.

### Mosaic Patterns are more Coarse-grained in the Adult *Pax6^+/−^* Corneal Epithelium than WT

**Figure 4:**
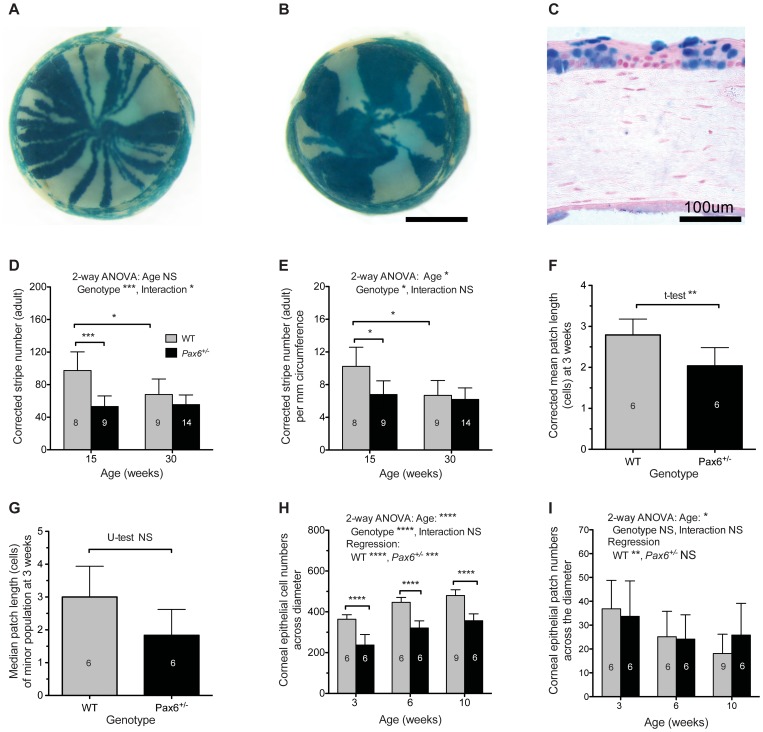
Analysis of mosaic patterns in the corneal epithelium of adult and 3–10 week old WT and *Pax6^+/−^* X-inactivation mosaics. (**A**) Adult WT *XLacZ^+/−^* mosaic eye showing characteristic radial stripes in the corneal epithelium. (**B**) Adult *Pax6^+/−^ XLacZ^+/−^* mosaic eye showing disrupted patterns with larger, more irregular patches. (**C**) Section through cornea of a 3-week old *XLacZ^+/−^* mosaic mouse eye showing β-gal positive and β-gal negative patches that were measured in the basal corneal epithelium. (**D, E**) Corrected stripe numbers per circumference (D) and corrected stripe numbers per mm of corneal circumference (E) for corneal epithelia from adult WT and *Pax6^+/−^,* X-inactivation mosaics at 15 and 30 weeks. Statistical comparisons were made by 2-way analysis of variance (ANOVA) followed by pairwise Bonferroni post-hoc tests. (**F, G**) Corrected mean patch lengths (in cell numbers across the corneal diameter of the basal corneal epithelium) for the β-gal positive cell population (F) and median patch lengths for the minor cell population (G) in WT and *Pax6^+/−^* mosaics at 3 weeks. Statistical comparisons were made by Student’s *t*-test with Welch’s correction (F) and Mann-Whitney U-test (G). (**H, I**) Mean numbers of cells (H) and patches (I) across the corneal diameter of the basal corneal epithelium in WT and *Pax6^+/−^* mosaics at three ages. Statistical comparisons were made by 2-way ANOVA followed by pairwise Bonferroni post-hoc comparisons of genotypes and separate linear regression analyses of ages for WT and *Pax6^+/−^* genotypes. Abbreviations: WT, wild-type; NS, not significant; *P<0.05; **P<0.01; ***P<0.001; ****P<0.0001. In D and E, corrected stripe numbers were calculated for both eyes and the mean value for each mouse was used for analysis. In F-I, the central histological section of one eye per mouse was analysed. The numbers of eyes per group is shown within the bars. Error bars are 95% CI. Scale bars: A,B = 1 mm; C = 100 µm.

As noted in the Introduction a previous study, showing that corrected stripe numbers were lower in *Pax6^+/−^*, *XLacZ^Tg/−^* than WT, *XLacZ^Tg/−^* mosaic corneas at 15 weeks [22], was used to predict that LESCs were reduced in number or qualitatively defective in *Pax6^+/−^* eyes. We confirmed these trends, using a semi-automated quantification method [40]. The striping patterns in *Pax6^+/−^*, *XLacZ^+/−^* mosaics were often more disrupted than in WT, with fewer larger patches that frequently did not extend radially from the limbus to the centre of the cornea, suggesting that cell movement was abnormal (Fig. 4A,B). The semi-automated analysis (Fig. 4D,E) confirmed previous results obtained by manual quantification [22]. In WT *XLacZ^+/−^* corneas, the corrected stripe numbers declined between 15 and 30 weeks whereas, in *Pax6^+/−^*, *XLacZ^+/−^* corneas, corrected stripe numbers were significantly reduced at 15 weeks but did not decline further between 15 and 30 weeks (Fig. 4D). These differences remained significant when the corrected stripe numbers were expressed per mm of corneal circumference to correct for the smaller size of *Pax6^+/−^* eyes (Fig. 4E). These results show that the mosaic pattern is more coarse-grained (fewer and wider stripes) in *Pax6^+/−^* than WT mosaic corneas and that in WT mosaic corneas the pattern becomes coarser between 15 and 30 weeks. The difference between *Pax6^+/−^* and WT mosaic patterns at 15 weeks could be explained by either reduced LESC numbers (hypotheses 1 in Fig. 1E–H) or reduced cell mixing during development (hypothesis 2 in Fig. 1I–L).

### Mosaic Patterns are not more Coarse-grained *Pax6^+/−^* Corneal Epithelium than WT at 3 Weeks

To test whether cell mixing is reduced during development of the ocular surface in *Pax6^+/−^* mice (hypothesis 2 in Fig. 1I–L), we measured β-gal-positive patch lengths in the basal corneal epithelium in histological sections of *Pax6^+/−^* and WT X-inactivation mosaic eyes (Figs. 4C,F,G), to compare cell mixing at three weeks, which is before the stripes emerge [24], [25]. The extent of cell mixing during development and growth of a mosaic corneal epithelium will affect the sizes of the coherent clones of contiguous β-gal-positive cells before the radial stripes emerge. The size of each β-gal-positive patch observed will depend on both the size of the constituent coherent clones and the number of adjacent coherent clones in the patch. For a one-dimensional string of β-gal-positive and β-gal-negative basal corneal epithelial cells in a tissue section, the mean number of β-gal-positive coherent clones per patch of β-gal-positive cells can be estimated as 1/(1–p), where p is the proportion of β-gal-positive cells [41], [42], [43]. The observed mean β-gal-positive patch length (mean cells per patch) was corrected by dividing it by 1/(1–p), to derive the ‘corrected mean patch length’, which is an estimate of the mean coherent clone length [43], [44].

The corrected mean patch length (calculated for β-gal positive patches) and the uncorrected median patch length calculated for the minority cell population [45] were used to compare the extent of cell mixing in the corneal epithelia of different groups of X-inactivation mosaics at 3 weeks (before stripes emerge). Both were smaller in *Pax6^+/−^*, *XLacZ^+/−^* than WT, *XLacZ^+/−^* corneas at 3 weeks and this difference was statistically significant for the corrected mean patch lengths (Fig. 4F). Thus, before the emergence of stripes, the mosaic pattern is not more coarse-grained (with larger coherent clones) in the *Pax6^+/−^* corneal epithelium than in WT, as would be predicted if the difference in adult mosaic stripe patterns was attributable to differences in cell mixing during development (hypothesis 2 in Fig. 1I–L). Indeed, cells appeared to be more finely mixed, which may reflect the reported reduction in cell-cell adhesion between *Pax6^+/−^* cells or between *Pax6^+/−^* cells and the stroma [27]. We therefore rejected the reduced cell mixing hypothesis (hypothesis 2 in Fig. 1I–K) and investigated whether adult *Pax6^+/−^* corneal epithelia are maintained by fewer LESC clones than WT (hypothesis 1 in Fig. 1E–H).

### Activation of LESCs in the *Pax6^+/−^* Ocular Surface

Analysis of patch lengths in mosaic corneas also provides a means of investigating the transition from patches to stripes. Fig. 4H,I shows that, between 3 and 10 weeks, the mean basal cell number across the corneal diameter increases with age for WT, *XLacZ^+/−^* corneas whereas the mean patch number decreases, implying that the patches increase in length over this period. This is consistent with observations on whole mount preparations showing that stripes emerge from the periphery at or before 5 weeks as a consequence of LESC activation and replace the randomly orientated patches formed during development [24], [25]. Similar trends were found for *Pax6^+/−^*, *XLacZ^+/−^* corneas (Fig. 4H,I), although the decrease in patch numbers was less pronounced and not significant by linear regression analysis. Nevertheless, the significant increase in cell number across the *Pax6^+/−^* corneal epithelial diameter, coupled with a trend for a decrease in patch numbers, suggests that LESCs are probably also activated in *Pax6^+/−^* eyes at this time and that they produce clones of cells that can extend radially across the corneal diameter.

### Identifying the Limbus in WT and *Pax6^+/−^* Whole-mount Preparations

**Figure 5:**
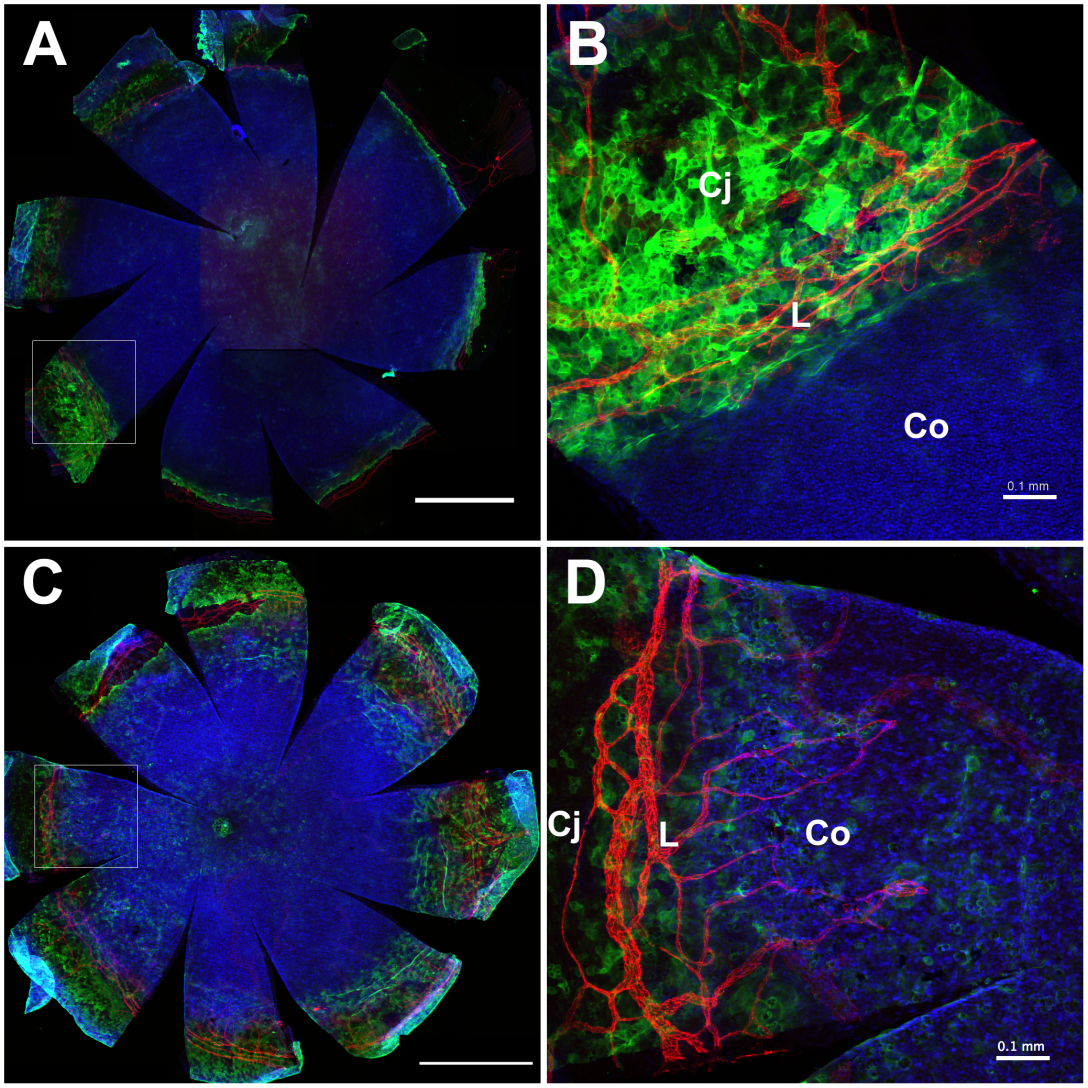
Identification of the corneo-limbal boundary in the WT and *Pax6^+/−^* mouse ocular surface epithelium. CD31 (red) and keratin 19 (green) double immunofluorescence staining in the ocular surface of (**A, B**) WT and (**C, D**) *Pax6^+/−^* mice. Images (B) and (D) are higher magnifications of the areas outlined in (A) and (C) respectively. Both CD31-positive blood vessels and keratin 19-positive epithelial cells are restricted to the conjunctiva and limbus in WT eyes but they both extend into the corneal epithelium in *Pax6^+/−^* mice. Abbreviations: L: Limbus; Co: Cornea; Cj: Conjunctiva. Red immunofluorescence: CD31; green: keratin 19; blue: TO-PRO3 iodide nuclear counterstain. Scale bars are 1 mm (A,C) and 0.1 mm (B,D).

Before testing whether limbal-LRCs were reduced in *Pax6^+/−^* eyes (predicted by hypothesis 1 in Fig. 1E–H), we characterised the boundary between the limbus and cornea in WT and *Pax6^+/−^* eyes by immunostaining for the blood vessel marker CD31 and keratin 19 (K19), which is present in the limbal and conjunctival epithelia but not the corneal epithelium [46]. Double K19/CD31 immunofluorescence of WT eyes (Fig. 5A,B) confirmed that both markers were restricted to the conjunctival and limbal epithelia and did not extend into the corneal epithelium, so identifying a clear corneo-limbal boundary. However, in *Pax6^+/−^* eyes, both K19 positive cells and, as previously reported [21], CD31-positive blood vessels extended into the cornea in *Pax6^+/−^* mice (Fig. 5C,D). Using TO-PRO3 as a nuclear counterstain, we were unable to identify nuclear morphological differences between limbal and corneal epithelial cells reported for whole-mount preparations counterstained with DAPI [10]. However, the K19 boundary in WT mice coincided with a morphological crease or in-folding that formed a ridge, which was apparent in nearly all the whole-mount WT specimens but less clear in *Pax6^+/−^* eyes (data not shown). This infolding has been described previously for WT mouse eyes [47], [48] and we used it as a marker to identify the limbus for analysis of BrdU label-retaining cells (LRCs).

### Limbal Label-retaining Cell Numbers are not Reduced in *Pax6^+/−^* Eyes or in Older WT Eyes

**Figure 6:**
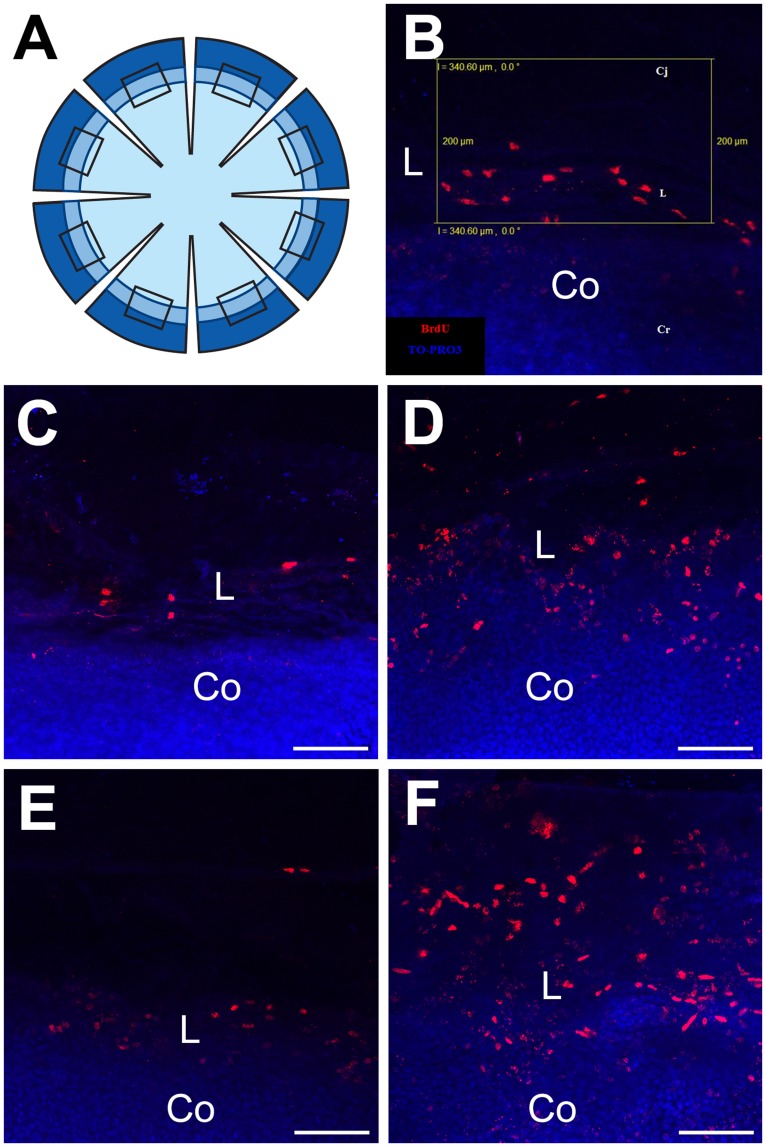
Identification of label-retaining cells in the limbal region of the WT and *Pax6^+/−^* ocular surface epithelium. (**A**) Diagram showing the 8 radial cuts in a corneal button, shaded to represent the cornea (lightest), limbus (intermediate) and conjunctiva (darkest). The rectangles show the location of the sampling boxes (one per sector but not to scale). (**B**) Rectangular 340×200 µm sampling box (yellow outline), used to count LRCs, superimposed on the limbal region of an image of BrdU-labelled nuclei (red) counterstained with TO-PRO3 iodide (blue) in a whole mount flattened corneal button with associated conjunctival tissue. (**C–F**) Examples of BrdU label-retaining cells in the limbal region after 1-week BrdU exposure and 10 week chase period in (**C**) 15-week old WT, (**D**) 15-week old *Pax6^+/−^*, (**E**) 30-week old WT and (**F**) 30-week old *Pax6^+/−^*. Pixel resolution: 1024×1024. Abbreviations: Cj: Conjunctiva; L: Limbus; Co: Cornea. Red immunofluorescence: BrdU; Blue: TO-PRO3 iodide counterstain. Scale bars are 100 µm.

Having rejected reduced cell mixing during development of the *Pax6^+/−^* ocular surface (hypothesis 2 in Fig. 1I–K) as an explanation of the reduced stripe numbers in adult *Pax6^+/−^* mosaic corneas, we wanted to test whether the adult *Pax6^+/−^* corneal epithelium is maintained by fewer LESCs than normal (hypothesis 1 in Fig. 1E–H). Results shown in Fig. 4D,E and previous work [22], [24], [25] also predicted a decline in LESC clone numbers in WT mice between 15 and 30 weeks. As most LRCs are thought to be putative stem cells, we compared LRC numbers in WT and *Pax6^+/−^* at both these ages. We exposed 15- and 30-week old WT and *Pax6^+/−^* mice to BrdU for 7 days and identified LRCs in the limbal region of eight corneal sectors (Fig. 6A). LRCs were identified by confocal microscopy and counted in a 340×200 µm sampling box superimposed over the limbal region in each sector (Fig. 6B) for each of the four groups of mice (Fig. 6C–F).

**Figure 7:**
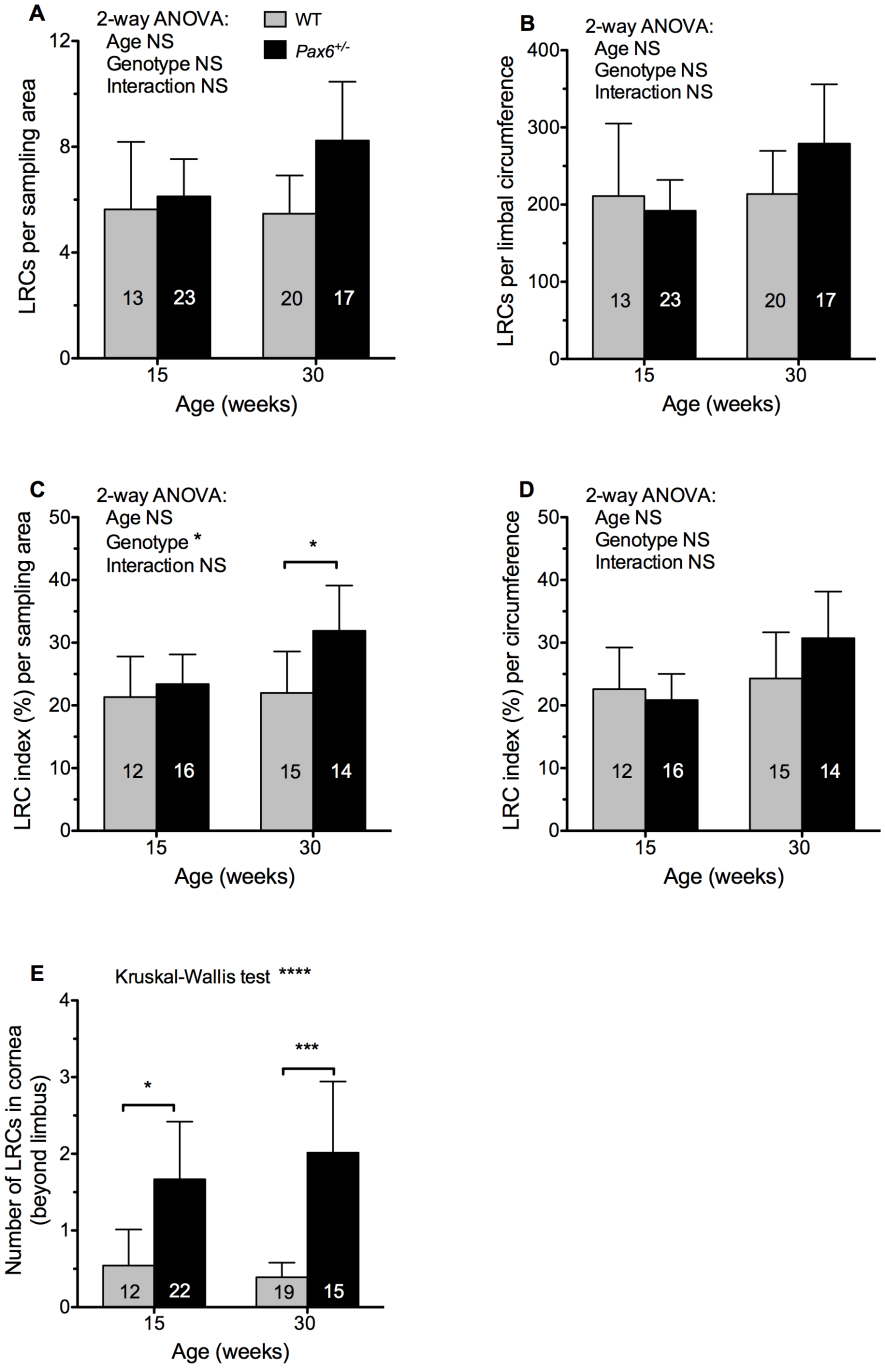
Comparison of label-retaining cell numbers between WT and *Pax6^+/−^* limbal and corneal epithelia. (**A**) Label-retaining cells (LRCs) per 340×200 µm sampling area. (**B**) LRCs per 200 µm wide ring around the limbal circumference. (**C**) LRC index per sampling area. (**D**) LRC index per limbal circumference. See text for explanation of LRC index. (**E**) LRCs that were within the cornea rather than the limbal area (more central than the sampling box shown in Fig. 6B). Results were compared by 2-way ANOVA followed by Bonferroni post-hoc tests (A–D) or Kruskal-Wallis test followed by Dunn’s multiple comparison test (E). Significant pairwise differences for genotypes or ages are shown by asterisks: *P<0.05, ***P<0.001, ****P<0.0001, NS, not significant. Error bars are 95% confidence intervals. The number of eyes per group is shown within each bar.

The mean number of LRCs per sampling box in the limbal region did not differ significantly between *Pax6^+/−^* and WT at either 15 or 30-weeks of age and did not differ between ages for either genotype (Fig. 7A). As *Pax6^+/−^* eyes are smaller than WT eyes we also estimated the number of LRCs within a 200 µm wide ring (the sampling box width) around the whole limbal circumference. Again, there were no significant differences among the four groups by 2-way ANOVA (Fig. 7B).

To reduce the effect of variation among individual immunofluorescence experiments, we also calculated an “LRC index” for each eye as described in the Materials and Methods. This normalised the results for each experiment and expressed them as a percentage value, such that the sum of the mean LRC indices for each of the four groups compared in each experiment was equal to 100%. The LRC index was significantly higher for *Pax6^+/−^* than WT eyes at 30 weeks when expressed per unit area but not at 15 weeks (Fig. 7C). However, when expressed as the LRC index per limbal circumference, the difference at 30 weeks was no longer significant (Fig. 7D). This implies that LRCs were significantly more frequent per unit area of limbus in *Pax6^+/−^* eyes than WT eyes at 30 weeks but because *Pax6^+/−^* eyes are smaller the total estimated number of limbal LRCs per eye is not significantly greater. None of the comparisons shown in Fig. 7A–D revealed a significant difference between ages for either genotype.

LRC cells were counted per unit area without counting BrdU-negative cells so we also compared the total cell packing densities in the ocular surface of the four groups. For technical reasons, the packing density was evaluated in the central cornea rather than the limbus. The mean cell numbers (±95% CI) per 150×150 µm sized sampling box in 3 fields from 3 eyes per group were estimated as 279±41 cells for 15-week WT, 207±45 for 15-week *Pax6^+/−^*, 266±117 for 30-week WT and 252±47 for 30-week *Pax6^+/−^*. A 2-way ANOVA revealed no significant differences between genotypes or ages. Thus, there was no evidence that the greater LRC index per unit area seen in *Pax6^+/−^* eyes compared to WT at 30 weeks (Fig. 7C) was a consequence of greater cell packing density in the *Pax6^+/−^* ocular surface in the older age group. Therefore, the only difference between *Pax6^+/−^* and WT genotypes observed, suggests that the number of limbal LRCs may be higher in some *Pax6^+/−^* mice, not lower as predicted if LESC numbers were reduced (hypothesis 1 in Fig. 1E–H).

### LRCs also Occur in *Pax6^+/−^* Corneas and are Associated with Blood Vessels

During the analysis of LRCs in the *Pax6^+/−^* limbus it was noted that there were also some LRCs in the *Pax6^+/−^* cornea itself (more central than the sampling box). The images used for counting limbal LRCs were also used to count corneal LRCs so this analysis was restricted to the peripheral areas of the cornea. A few corneal LRCs were identified and counted in WT eyes but, if the limbal sampling box was imprecisely positioned over the limbus, these could have been limbal LRCs. Significantly more corneal LRCs were identified in *Pax6^+/−^* corneas than in WT corneas at both ages (Fig. 7E).

**Figure 8:**
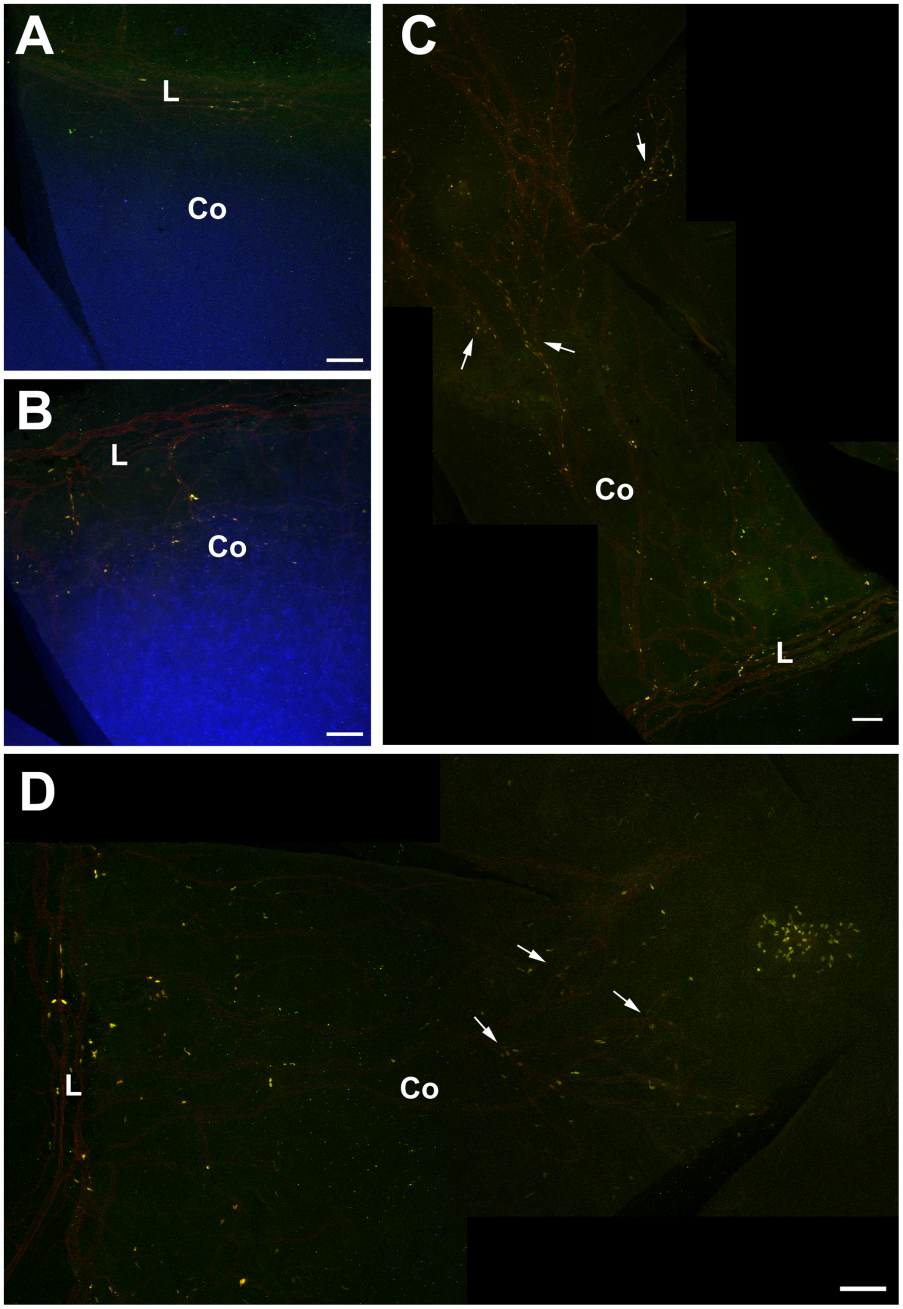
Association of BrdU label-retaining cells with blood vessels in *Pax6^+/−^* corneas. Double immunofluorescent detection of BrdU (yellow) and CD31-positive blood vessels (red) in WT and *Pax6^+/−^* mice. (**A**) Flattened WT cornea showing BrdU-positive, label-retaining cells (LRCs) and CD31-positive blood vessels in the limbus. (**B**) Flattened *Pax6^+/−^* cornea demonstrating CD31-positive blood vessels extending from the limbus into the cornea and BrdU LRCs in the cornea. (**C, D**) Montages of flattened *Pax6^+/−^* corneas showing CD31-positive blood vessels and BrdU LRCs even in the central cornea. Arrows show blood vessels with adjacent BrdU-positive cells (LRCs). For demonstration purposes the counterstain channel was deactivated. Abbreviations: L: Limbus; Co: Cornea; Yellow immunofluorescence: BrdU. Red immunofluorescence: CD31; Blue: TO-PRO3 iodide counterstain. Scale bars are 100 µm.

The identification of LRCs in the corneal epithelium of *Pax6^+/−^* eyes in conjunction with the observation that blood vessels invade the corneal epithelium in *Pax6^+/−^* mice (Fig. 5C,D) prompted a study to localise both LRCs and blood vessels in the same samples. WT and *Pax6^+/−^* mice were prepared for LRC analysis at 15 weeks (1-week BrdU; 10-week chase) and eyes were immunostained for BrdU and CD31 blood vessels. The distribution of LRCs appeared very similar to the blood vessels (Fig. 8). In WT eyes, LRCs were restricted to the limbus (Fig. 8A) but in *Pax6^+/−^* eyes they were present in both the limbus and cornea (Fig. 8B) and usually associated with blood vessels (Fig. 8C,D). The fluorophores that were used did not provide a suitable colour combination to determine whether BrdU staining (yellow) co-localised with CD31-positive blood vessels (red). However, this observation suggests that the BrdU-LRCs in *Pax6^+/−^* eyes are not all LESCs.

## Discussion

### Cell Turnover in the Wild-type Mouse Corneal Epithelium

The decline in BrdU in the WT basal corneal epithelium after 24 hours, as cells moved vertically (apically) to the suprabasal layers, was followed by a decline in labelled suprabasal cells, after 3 days. This showed that most cells labelled in the basal layer were lost from the corneal epithelium by day 14 and implies that the corneal epithelium ‘turnover time’ is ≤14 days. However, most BrdU-positive cells will survive for less than this, once they leave the basal layer, because some will remain in the basal layer for several cell generations. The decline of labelled suprabasal cell numbers between 3 and 7 days suggests that some basal cells are shed from the surface within 7 days of labelling. This is similar to previous turnover time estimates of 6–7 days in mice [49], 3^1^/_2_–4 days, 12.3 days or 2 weeks in rats [49], [50], [51] and 9–10 days or >14 days in rabbits [52], [53].

The turnover time is shorter than the time required for LESCs to replace the corneal epithelium. This has been termed the ‘renewal time’ [52] and estimated as 7 weeks for adult mice, [5] and 9–12 months in rabbits [52].

For WT corneas, there was little evidence of regional differences in cell proliferation or movement to the suprabasal layers. Results of previous studies are inconsistent [50], [53], [54], [55], [56], [57]. Some differences among studies may reflect species or technical differences and others may be explained by circadian rhythms that affect corneal epithelial proliferation in mice, rats and rabbits [55], [56], [58] and may affect proliferation differently at the corneal periphery and centre [55].

### Increased Cell Turnover in the *Pax6^+/−^* Mouse Corneal Epithelium

A previous BrdU study showed significantly higher labelling in the basal *Pax6^+/−^* corneal epithelium compared to WT [28]. Our results showed a similar trend but it was not significant. The present study is the first to compare the distributions of BrdU-labelled cells separately in the basal and suprabasal layers of *Pax6^+/−^* and WT corneas. This demonstrated that relatively more labelled cells move from the basal to the suprabasal layers within the first 24 hours of labelling in *Pax6^+/−^* than WT corneas and the difference is greatest in the central cornea. As this vertical cell movement is irreversible and is the beginning of cell loss, we conclude that greater corneal epithelial cell loss occurs in *Pax6^+/−^* than WT corneas. Overall, this shows that *Pax6^+/−^* corneal epithelial cell turnover is faster because basal cells both proliferate more frequently [28] and are more readily lost from the cornea than WT basal cells. Furthermore, the greater cell loss from the *Pax6^+/−^* central corneal epithelium may be because the *Pax6^+/−^* cornea is more fragile and the central region is most vulnerable to injury.

### Limbal Epithelial Stem Cell Function in Older WT Mice

We used LRCs to identify putative LESCs but this is not specific for stem cells. If both actively proliferating and relatively quiescent stem cell populations exist, as they do in some other tissues, only the quiescent ones will be identified as LRCs [59], [60], [61] and only those that divide during the labelling period will be included. Furthermore, any somatic cells that divide during the labelling period but subsequently undergo terminal differentiation before diluting the label significantly will also be identified as LRCs.

The presence of LRCs in the limbus but not the central cornea in WT mice is consistent with the conventional view that the adult corneal epithelium is maintained by slow-cycling LESCs, some of which are identified as label-retaining cells (LRCs). However, the quantitative LRC results failed to support two predictions prompted by analysis of radial mosaic corneal epithelial stripe patterns. There was no evidence that LRC numbers declined between 15 and 30 weeks in WT mice and no evidence that *Pax6^+/−^* mice had fewer LRCs than WT mice at 15 weeks. The mosaic analysis compares corrected stripe numbers, which relates to the numbers of active coherent clones of stem cells (comprising 1 or more stem cells) rather than the numbers of individual stem cells. Thus, stripe numbers depend both on numbers of stem cell clones (≤ number of stem cells) and the ability of the stem cells to function (generate a stripe of TACs in the corneal epithelium). Different results may be obtained for the mosaic and LRC analyses for two reasons. First, LESC function may change in a way that affects stripe numbers and LRC numbers differently. Second, LESC function may not change with age but stripe numbers could decline for an unrelated reason.

In the first case, several age-related changes could affect LESCs and cause stripe numbers to change independently of LRC numbers. (a) LESCs in older mice may continue to divide, and be detectable as LRCs, but more may be defective and unable to establish long-lived clones of corneal epithelial cells, identifiable as stripes in mosaic eyes. (b) Age-related changes in LESC cell cycle kinetics may cause longer or more frequent periods of quiescence, which reduce stripe production, but this might not reduce LRC numbers if, for example, reduced BrdU labelling was balanced by increased BrdU retention. (c) If the limbus harbours separate populations of actively proliferating stem cells (which produce most of the stripes) and more quiescent stem cells (detectable as LRCs) the age-related change could be restricted to stripe numbers if only the more active stem cell population decreased with age. (d) Both stripe numbers and LRCs produced by LESCs might decline with age but if the cell cycle slowed in some other cell types these might also be identified as LRCs and the overall LRC number would not decline in parallel with the stripe numbers.

In the second case, an age-related decline in corrected stripe number might reflect a decline in numbers of active LESC *clones* without any reduction in the numbers of active stem cells or their function if stochastic neutral drift in LESC populations eliminated some clones and expanded others. This has been suggested as an explanation of the coarsening of mosaic patterns derived from stem cell clones in the mouse testis [62] and mouse intestine [63], [64], [65] and could also apply to the corneal stripes as discussed elsewhere [66].

### Limbal Epithelial Stem Cell Function in *Pax6^+/−^* Mice

There was no evidence to support the prediction from the stripe analysis that *Pax6^+/−^* mice had fewer LRCs than WT mice at 15 weeks. Moreover, the limbal LRC index per unit area was significantly higher (not lower) for *Pax6^+/−^* than WT eyes at 30 weeks (Fig. 7C). There were also significant numbers of LRCs within the cornea itself (Fig. 7E). These results are consistent with two possibilities.

First, *Pax6^+/−^* eyes could have at least as many slow-cycling stem cells in the limbal epithelium as WT mice plus an additional population of slow-cycling stem cells in the corneal epithelium itself. The presence of LRCs in the corneal epithelium has also been reported for *Dstn^corn1/corn1^* mice [67]. In this case there was no evidence of centripetal cell movement and the authors suggested that the mutant corneal epithelium was maintained by stem cells within the cornea itself. Cell movement may be abnormal in *Pax6^+/−^* corneas [68], [69] but there is no evidence it has ceased. Therefore, if the *Pax6^+/−^* corneal LRCs represent stem cells, they could have arisen within the cornea or moved into the cornea from the limbus or conjunctiva. However, our LRCs study provides no evidence for stem cells within the corneal epithelia of WT mice as proposed to explain the results of limbal transplantation experiments [70].

If the *Pax6^+/−^* corneal LRCs were stem cells, their apparent association with blood vessels (Fig. 8) is consistent with other reports of close relationships between putative stem cells, identified as LRCs, and blood vessels in different tissues [71], [72], [73], [74], [75]. The trend for *Pax6^+/−^* eyes to have more LRCs than WT, particularly at 30 weeks (Fig. 7) also raises the possibility that an increasing demand for cells to maintain the corneal epithelium in this model of chronic corneal wound healing produces higher numbers of LESCs in *Pax6^+/−^* heterozygotes (e.g. by symmetrical LESC division) so *Pax6^+/−^* LESC numbers might actually increase with age. However, the increase in LRCs between 15 and 30 weeks was not statistically significant and there is no other evidence for an age-related increase in *Pax6^+/−^* LESCs.

The second possibility is that some of the LRCs in the *Pax6^+/−^* limbus, and perhaps all of those in the cornea, are other types of cells that divided during the BrdU exposure period but then dropped out of the cell cycle. The close association between corneal LRCs and blood vessels in *Pax6^+/−^* corneas suggests that some LRCs may actually be vascular endothelial cells or their associated pericytes, immediately underlying the epithelium, which proliferated during the period of BrdU exposure and then terminally differentiated. Moreover, the LRC index per unit area was only significantly greater in *Pax6^+/−^* than WT eyes in the older age group (Fig. 7C), which is when more *Pax6^+/−^* corneas are likely to show neovascularisation. In addition, some *Pax6^+/−^* corneal LRCs could be inflammatory cells that phagocytosed labelled cells, as reported for activated macrophages [76], [77]. We, therefore, conclude that our limbal LRC analysis is unlikely to only identify stem cells in the *Pax6^+/−^* ocular surface so does not accurately represent the relationship between *Pax6^+/−^* and WT LESC numbers. The LRC approach has not provided an adequate means of testing whether *Pax6^+/−^* mice have a LESC deficiency, so alternative approaches are required.

### Abnormal Maintenance of the Corneal Epithelium in *Pax6^+/−^* Mice

Although our results do not demonstrate whether LESCs are depleted or defective in *Pax6^+/−^* mice, they have provided further insights about the abnormal maintenance of the *Pax6^+/−^* corneal epithelium. We confirmed that the *Pax6^+/−^* corneo-limbal boundary is indistinct, which is consistent with the widely-accepted notion that conjunctiva intrudes into the mouse *Pax6^+/−^* and human *PAX6^+/−^* cornea because there is a LESC deficiency. However, this is not proof of LESC deficiency and other explanations remain possible [28]. For example, a recent study shows that when goblet cells are present in the corneal epithelium they need not originate from the conjunctiva [78].

Our evidence that cell loss is greater than normal in the *Pax6^+/−^* corneal epithelium is consistent with previous evidence for greater corneal epithelial fragility, attributed to abnormal expression of molecules important for cell adhesion [27], [28], [29]. In the WT corneal epithelium, cell loss may be driven by a combination of factors, possibly including basal cell overcrowding, as reported for the *Drosophila* notum [79]. In the *Pax6^+/−^* corneal epithelium, it seems likely that the reverse applies, such that greater epithelial fragility causes increased cell loss, which drives increased production of TACs.

To ensure the corneal epithelium is maintained at a uniform thickness, cell production, movement and loss must be co-ordinated and balanced. For WT corneas this balance was originally described in terms of the X, Y, Z hypothesis [80]. Updated as the limbal stem cell hypothesis, this can be restated as Y_SC_+X_TAC_ = Z_L_ [66] where Y_SC_ denotes production of basal corneal epithelial cells by LESCs, X_TAC_ denotes the proliferation of corneal epithelial TACs, and Z_L_ denotes epithelial cell loss from the corneal surface. In the *Pax6^+/−^* corneal epithelium, cell loss is high and may exceed cell production. TAC proliferation is also high [28] but we still do not know whether TAC production by LESCs is low, so maintenance of the *Pax6^+/−^* corneal epithelium may be summarised provisionally as Y_SC_^(?)^+X_TAC_^high^ = Z_L_^high^ if corneal homeostasis is in equilibrium or Y_SC_^(?)^+X_TAC_^high^<Z_L_^high^ if corneal epithelial cell numbers decline (excluding other encroaching cell types). Corneal epithelial TACs might be stimulated to be more proliferative in response to greater cell loss from a fragile corneal epithelium and/or to compensate for deficient or defective LESCs. This could involve decreasing cell cycle times, increasing the number of TAC divisions beyond the normal maximum or changing the balance of cell divisions so more TACs fulfil their maximum proliferative potential, as may occur in response to wounding [6].

The corneal epithelium of *Pax6^+/−^* mice is already thin by E18.5, just before birth [21], implying that this is a developmental abnormality. If homeostasis is initially quantitatively normal, the corneal epithelium might be maintained adequately for some time, although it will be thinner than in WT mice. However, in adult *Pax6^+/−^* mice, it seems likely that increased corneal epithelial cell loss exceeds the cell production capacity and causes corneal homeostasis to become unstable, resulting in progressive corneal deterioration. This is supported by morphological evidence of cells sloughing off an irregular, vesiculated superficial *Pax6^+/−^* epithelial layer [21]. In this study the number of epithelial cell layers was reduced from 4–5 cell layers in WT to 2–4 in *Pax6^+/Sey-Neu^* heterozygotes and a similar difference was seen in the present investigation (compare WT in Fig. 2A,C with *Pax6^+/−^* Fig. 2B,D). However, an even greater reduction (from 8–10 cell layers in WT to 1–7 layers) was reported for both *Pax6^+/Sey-Neu^* and *Pax6^+/Sey-Dey^* heterozygotes on a different genetic background [27]. The extreme cases where the corneal epithelium was reduced to a single layer strongly suggest that corneal homeostasis had become quantitatively destabilised.

Qualitative corneal epithelial deterioration is likely, even if additional encroaching cells maintain total cell numbers adequately, because these will not be phenotypically identical to corneal epithelial stem cells and so corneal function will be compromised. A shortfall in corneal epithelial cell production may arise simply because the increased cell loss from the *Pax6^+/−^* corneal epithelium exceeds the renewal capacity of even a normal complement of LESCs. We suggest that hallmarks of corneal deterioration, including ‘conjunctivilisation’ and the appearance of goblet cells within the corneal epithelium, that are often taken as evidence of LESC-deficiency, might also occur in the absence of LESC deficiency if corneal homeostasis is destabilised by excessive cell loss.

There is also evidence that reduced Pax6 levels in the human *PAX6^+/−^* corneal epithelium is accompanied by a down-regulation of K12 [81]. This parallels the situation described in the Introduction for *Pax6^+/−^* mice and indicates that corneal epithelial differentiation is abnormal. Reduced K12 in *PAX6^+/−^* aniridia also probably increases epithelial fragility, so contributing to the recurrent corneal erosions that are characteristic of human aniridia-related keratopathy [82]. This increased epithelial fragility suggests that cell loss is likely to be abnormally high in people with *PAX6^+/−^* aniridia as well as heterozygous *Pax6^+/−^* mice. These observations indicate that the effect of Pax6 depletion on the human corneal epithelium is not restricted to the LESCs so the abnormal phenotype associated with ARK is probably not mediated entirely via LESC-deficiency.

### Conclusion

Quantitative LRC experiments did not support the prediction that LESCs decline with age in WT mice and failed to test adequately whether *Pax6^+/−^* mice have a LESC-deficiency. Several possible explanations are discussed that require further investigation. Although it remains unclear whether *Pax6^+/−^* mice have LESC-deficiency, our BrdU-labelling analysis implied that epithelial cell turnover is faster than normal and this is probably driven by increased cell loss. Excessive cell loss might destabilise corneal homeostasis and cause *Pax6^+/−^* corneal epithelial deterioration, even in the absence of LESC-deficiency. This also raises the possibility that excessive cell loss could also play a causal role in human aniridia-related keratopathy.
